# Dysplasia epiphysealis hemimelica combined with contralateral accessory scaphoid bone: A case report and literature review

**DOI:** 10.1097/MD.0000000000017887

**Published:** 2019-11-11

**Authors:** Bo Li, Jie Wen, Hong Liu, Sheng Xiao, Xin Li, Ke Fang, Ming Zeng, Zhongwen Tang, Shu Cao, Bo Lee, Fanling Li

**Affiliations:** Department of Pediatric Orthopedics, Hunan Provincial People's Hospital, the First Affiliated Hospital of Hunan Normal University, Changsha, Hunan, China.

**Keywords:** accessory scaphoid, children, dysplasia epiphysealis hemimelica, foot pain

## Abstract

**Rationale:**

Dysplasia epiphysealis hemimelica (DEH), also known as Trevor disease, is a rare, developmental bone disorder of childhood.

**Patient concerns:**

A 9-year-old girl was admitted due to pain in front of the medial malleolus of her right foot after a long walk or distance movement, in which the pain could be relieved after rest, while it was repeated and lasted for several months.

**Diagnosis:**

Dysplasia epiphysealis hemimelica

**Interventions:**

The patient underwent an open resection surgery. After operation, the pain was totally relieved. Postoperative pathological diagnosis showed DEH.

**Outcomes:**

At the 6-month follow-up, pain and claudication symptoms fully disappeared, and range of motion of the right foot returned to normal level.

**Conclusions:**

Dysplasia epiphysealis hemimelica is an uncommon disease which can cause pain of foot in children.

**Lessons:**

When the pediatric orthopedic surgeon treated the children suffered with foot pain should be aware of this rare disease, especially accessory scaphoid bone was found in another foot.

## 1 Introduction

Dysplasia epiphysealis hemimelica (DEH), also known as Trevor disease, is a rare, developmental bone disorder of childhood. It is characterized by an abnormal overgrowth of cartilage arising from the cartilage, which is normally present in the terminal ends (epiphyses) of the long bones.^[1]^ To date, the etiology of DEH has still remained elusive. Lower extremities are often involved, with knee and ankle joints. DEH is common in unilateral limb, especially more frequent in the right side compared with the left side, as well as being the most common in the medial side. DEH often occurs between the age of 2 and 14 years, the incidence of DEH is about 1:1,000,000, and that is also further prevalent in males, with a male-to-female ratio of about 3:1.^[1–4]^ The purpose of this study was to report a 9-year-old girl with DEH in the right talus combined with accessory scaphoid bone in her left foot.

## 2 Case report

**Figure 1:**
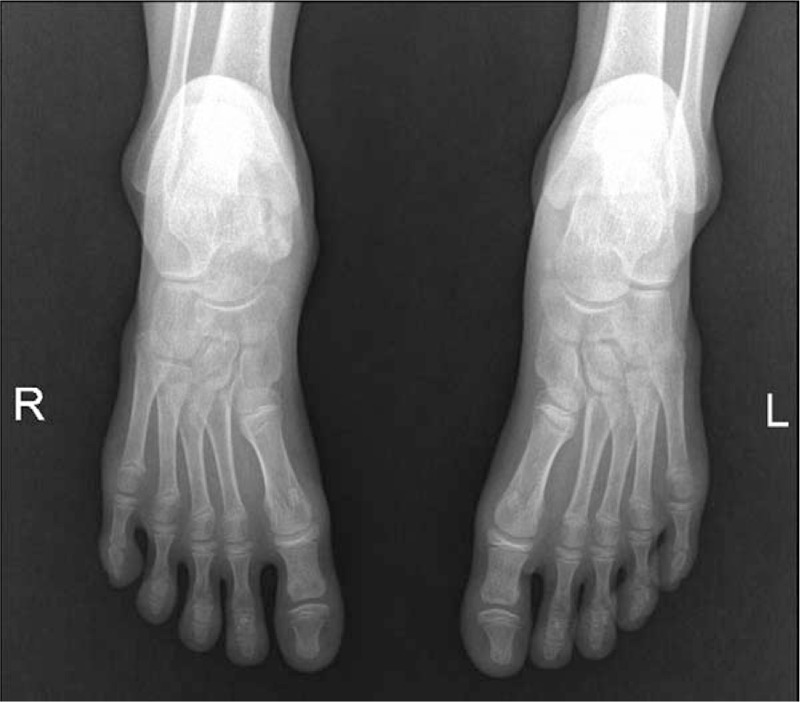
Anteroposterior X-ray view and lateral film of the feet showed a bone mass in the medial talus, with a smooth edge. The accessory scaphoid bone can be observed in the left foot.

**Figure 2:**
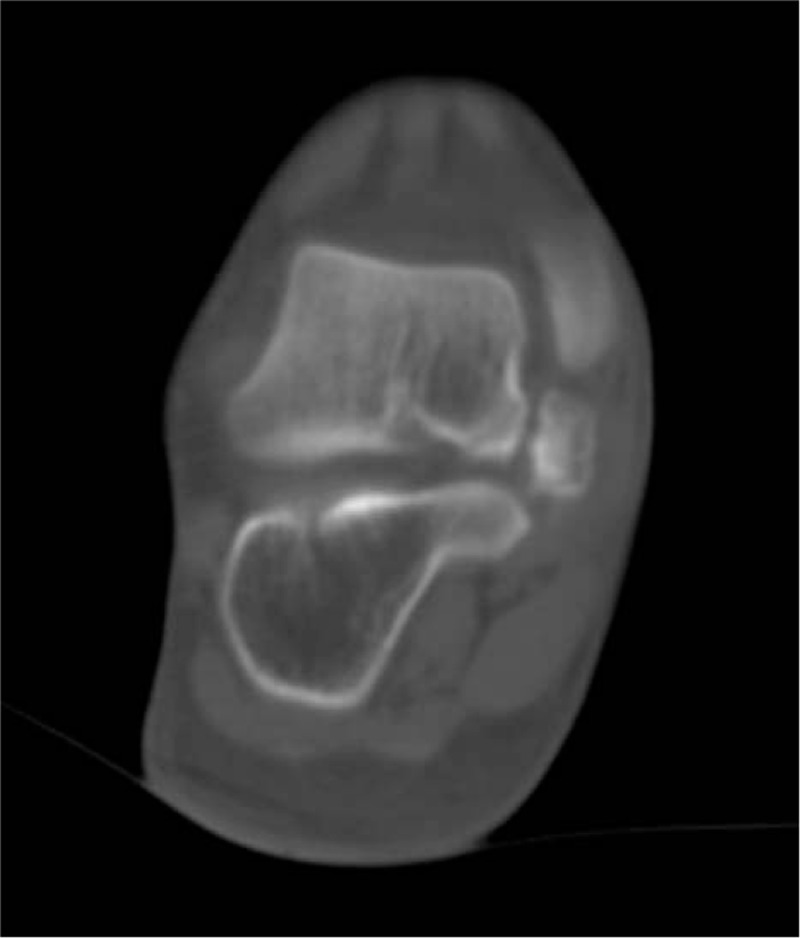
CT showed a bone mass in the talus process and medial talus of the right foot root, with uneven bone density and obvious sclerosis, and a pseudo-joint can be observed between the bone mass and the talus.

**Figure 3:**
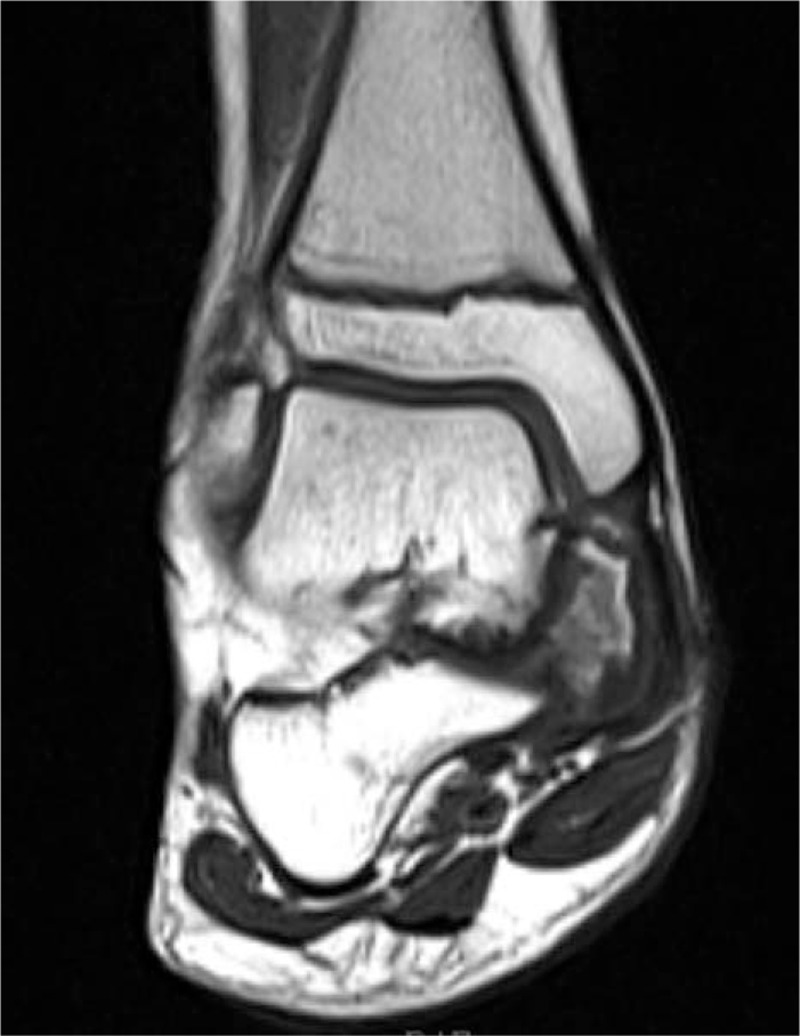
MRI showed a free mixed signal lesion and obvious sclerosis in the talus process and medial talus of the right foot root.

**Figure 4:**
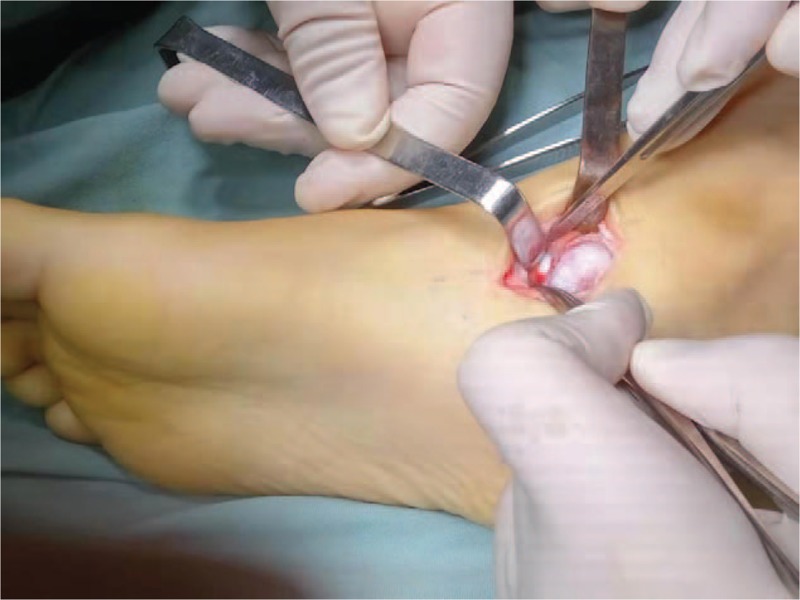
During operation, the bone mass was located at the back of scaphoid bone, and the medial part of talus was osseous, that formed articular surface with talus and calcaneus, and covered with cartilage.

**Figure 5:**
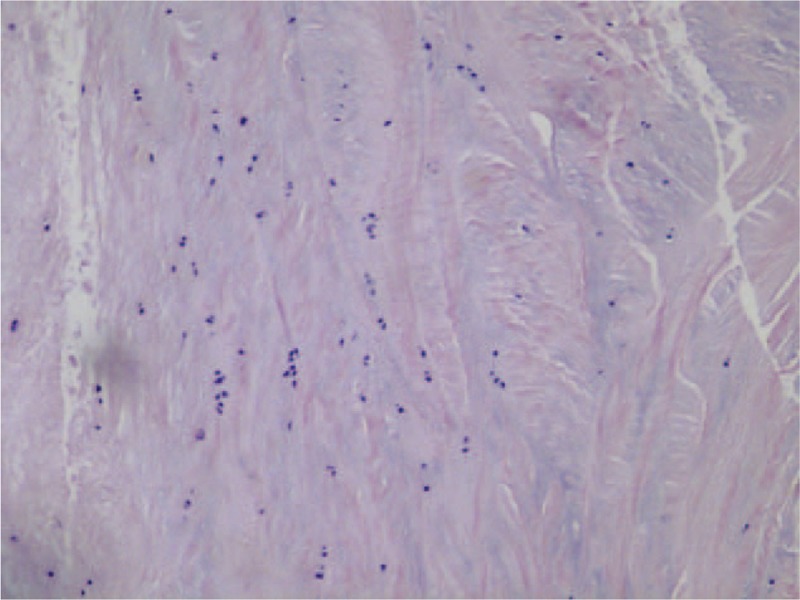
Pathological section showed a large number of fibroblasts, cartilage tissue, and no heterocysts.

A 9-year-old girl complained of pain in front of the medial malleolus of her right foot after a long walk or distance movement, in which the pain could be relieved after rest, while it was repeated and lasted for several months. There was no obvious history of trauma in the child. Physical examination revealed a mass in front of the medial malleolus of her right foot, which protruded from the skin that had a hard touch, clear margin, and can be propelled as well. There was no obvious motor dysfunction in her right foot, and no remarkable abnormality was noted in the examination of neurological function. X-ray imaging (Fig. 1) showed a bone mass in the medial talus, with a smooth edge. Computed tomography (CT) scan of her right foot (Fig. 2) revealed that there was a bone mass in the talus process and medial talus of her right foot root; the bone density was uneven and the sclerosis was obvious; a pseudo-joint can be observed between the bone mass and the talus. Magnetic resonance imaging (MRI) (Fig. 3) illustrated that there was a free mixed signal lesion and obvious sclerosis in the talus process and medial talus of the right foot root, leading to formation of the pseudo-joint between the right foot root and the talus. In the surgical treatment, supine position was adopted, and a dorsal medial incision of right ankle was made to separate tissue layer-by-layer, and then, a mass near the talus could be observed, with a size of 2 × 2 × 2 cm^3^ (Fig. 4). It was osseous and formed articular surface with talus and calcaneus. The surface of the mass was covered with cartilage, and the osseous mass was fully removed as well. After surgery, the ankle joint was fixed with plaster cast. Postoperative pathological diagnosis confirmed DEH (Fig. 5).

**Figure 6:**
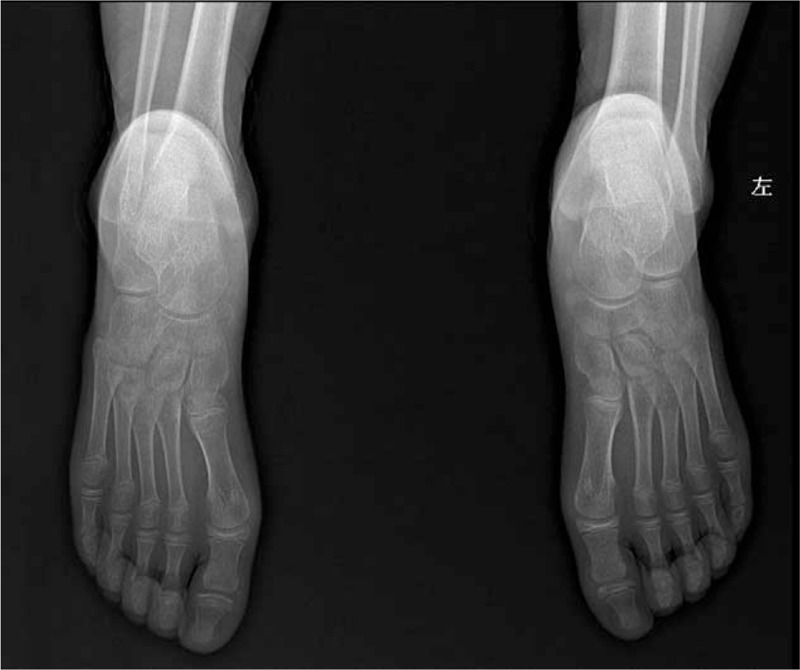
Followed X ray after 6 months shows the mass was resect totally and accessory scaphoid bone in her left foot remain still.

After surgery, plaster cast was worn for 6 weeks and functional exercises were performed after removal of plaster cast. After 6 months of follow-up, the pain and claudication symptoms fully disappeared, and range of motion (ROM) of the right foot returned to normal level. Medical X-ray imaging showed that the mass was fully resected and accessory scaphoid bone in her left foot could be visible (Fig. 6).

## 3 Discussion

Trevor disease or DEH refers to intra-articular epiphyseal osteochondroma. Although that was initially by Mouchet and Belot in 1926 and called “tarsomégalie”,^[5]^ however, it was delineated as a distinct entity by Trevor in 1950.^[1]^ In 1956, Fairbank et al described the characteristic involvement of characteristic of either the lateral or medial half of a single limb. He first used the term “DEH,” which appeared as the most logical and frequently used nomenclature.^[2]^ The pathological characteristics of DEH are mainly abnormal development of epiphyseal cartilage, leading to excessive proliferation and hypertrophy of epiphyseal chondrocyte, excessive ossification of epiphyseal cartilage, and multiple ossification centers. Therefore, DEH is taken as a kind of idiopathic and abnormal hyperplasia of epiphysis into account, which has no association with genetic and environmental factors.^[6]^

For DEH, the diagnostic criteria based on imaging were originally presented by Fairbank et al^[2]^ as follows:

(1) unbalanced growth of bone;
(2) ossification centers being unrelated to epiphysis.

X-ray imaging showed abnormal broadening of epiphysis in the lesion and multiple irregular ossification centers (especially in the early stage of DEH) in the bone mass, which were not connected with normal bone tissue; with the growth and development of secondary ossification centers, multiple ossification centers can be fused into a large ossification center.^[8]^ However, X-ray imaging plays a minor role and cannot accurately evaluate the size and shape of bone mass. However, CT images can clearly illustrate the size of bone mass and its relationship with normal epiphysis. In particular, when the shape and size of bone mass are irregular and large, three-dimensional CT reconstruction plays a substantial role in evaluation of surgery and selection of an optimal surgical method.^[9]^ However, CT images cannot show the composition of ossification center, and also cannot accurately assess the extent of surgical resection, while MRI appropriately shows the extension of osteochondral allograft transplantation for treatment of cartilage defects, as well as its relationship with surrounding tissues, playing a pivotal role in the evaluation of surgical resection extent.^[8,9,10]^

In addition, DEH mainly occurs in the femur, tibia, and talus. Azouz et al ^[7]^ first, classified this disease into three types according to the location of lesion distribution: limited type, involving only one epiphysis of lower extremity; classical type, involving multiple epiphyses of lower extremity; and systemic type, involving the entire epiphysis of lower extremity. The incidence of DEH in talus is rare. We have summarized the findings of previous studies concentrated on DEH in talus conducted during 1950 to 2018 (Table 1). The case reported in current study was a 9-year-old girl with DEH near the talus. The initial manifestation of DEH was pain at the ankle joint, which was obvious during walking and limited ankle movement. After about half a month from appearance of symptoms, the girl was diagnosed with DEH by physical and imaging examinations, which belonged to the limited type.

**Table 1:**
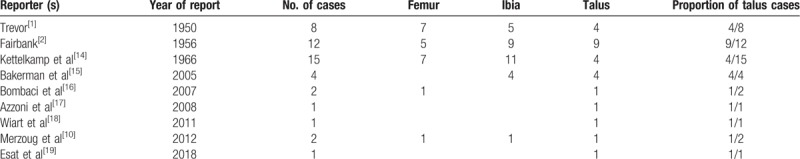
Cases of DEH in talus reported by previous studies.

Accessory scaphoid bone originates from the secondary ossification center of the scaphoid bone of foot. There is only one ossification center of the normal scaphoid bone of foot. The accessory scaphoid bone is congenital abnormality of the secondary ossification center of the scaphoid bone. The incidence of the disease in the general population is about 5% to 14%.^[20]^ At present, accessory scaphoid bone is a congenital abnormality of ossification center. Compared with the pathological characteristics of DEH, the pathological characteristics of accessory scaphoid bone are more prone to normal bone structure.

To date, a limited number of studies have concentrated on DEH combined with other bone structural abnormalities. Greenspan et al^[21]^ reported a 15-year-old DEH boy with mixed sclerosing bone dysplasia; Takegami et al^[22]^ presented an 8-year-old DEH boy with polysyndactyly; Graves et al^[23]^ reported a 6-year-old DEH boy with flat foot. The case reported in the present study is a 9-year-old girl with DEH in the talus medial of the right foot combined with accessory scaphoid bone in the left foot, that is the first case reported in English literature. In the diagnosis, as the location of DEH in this case is close to that of the accessory scaphoid bone, confusion may occur during diagnosis. In the course of consultation, the girl was also diagnosed with osteochondroma and accessory scaphoid in other hospitals. Eventually, she was pathologically diagnosed with DEH.

Osteochondroma typically occurs in the metaphysis of long bones and in older children. DEH originates from epiphysis and mainly occurs in younger children.^[17]^ The histological manifestations of DEH are very similar to those of chondroma. Besides, it is difficult to diagnose DEH by histology alone. However, Steven et al^[11]^ observed that the chondrocyte population of DEH was concentrated in chaotic, non-absorbable chondrocyte fragments and small ossification centers by comparing the pathological changes of DEH with osteochondroma, while osteochondroma was mainly accompanied by further mature ossification processes similar to the development of normal tissues. It also has been reported that EXT1 and EXT2 were found in osteochondroma by genetic examination, that may provide an appropriate evidence when two pathological examinations are similar.^[12]^

Conservative treatment of DEH contains a number of therapeutic effects, however, according to the existing reports, active surgical excision can achieve satisfactory results for DEH patients, especially in the early stage of the disease.^[1,3,4,8–11]^ In case reported in the present study, the lesion occurred in the medial talus, often rubbed with the shoes and caused repeated pain. Conservative treatment was also ineffective, and with the increase of the bone mass, the symptoms remarkably became serious. After surgical resection, the pain disappeared and the ROM of the joint returned to normal level after 6 months of follow-up. It should be noted that although there are no reports concerning malignant changes in DEH, however, DEH can be increased with the growth of bone, thus children should be followed-up until bone maturation. During follow-up MRI is of great significance to detect the recurrence of DEH.^[13]^

DEH is described as a type of over growth at one or more epiphyses. The case herein reported was a DEH patient with contralateral accessory scaphoid bone. Additionally, DEH should be considered in case of abnormal bone mass near the talus. Early surgical treatment can achieve satisfactory clinical efficacy as well.

## Author contributions

**Data curation**: Shu Cao, Bo Lee, Fanling Li.

**Methodology**: Jie Wen, Xin Li.

**Project administration**: Hong Liu.

**Software**: Ke Fang.

**Supervision**: Sheng Xiao.

**Visualization**: Ming Zeng, Zhongwen Tang.

**Writing – original draft**: Bo Li.

**Writing – review & editing**: Jie Wen, Sheng Xiao.
